# Measuring patient-reported outcomes: moving beyond misplaced common sense to hard science

**DOI:** 10.1186/1741-7015-9-86

**Published:** 2011-07-14

**Authors:** Stephen P McKenna

**Affiliations:** 1 Director of Research, Galen Research Ltd, Enterprise House, Manchester Science Park, Lloyd Street North, Manchester M15 6SE, UK

## Abstract

Interest in the patient's views of his or her illness and treatment has increased dramatically. However, our ability to appropriately measure such issues lags far behind the level of interest and need. Too often such measurement is considered to be a simple and trivial activity that merely requires the application of common sense. However, good quality measurement of patient-reported outcomes is a complex activity requiring considerable expertise and experience. This review considers the most important issues related to such measurement in the context of chronic disease and details how instruments should be developed, validated and adapted for use in additional languages. While there is often consensus on how best to undertake these activities, there is generally little evidence to support such accord. The present article questions these orthodox views and suggests alternative approaches that have been shown to be effective.

## Opinion

Questionnaires are ubiquitous throughout life these days. Medicine is no different, with the patient rightly seen as a client whose views are crucial to gaining a clear understanding of anything from the quality of service provision to treatment effectiveness. Patients are increasingly regarded as one of the key stakeholder groups in medicine that, alongside regulators, payers and clinicians, can influence access to and reimbursement for pharmaceutical products. Much of the information on patient views is collected via questionnaires. Many, if not most, of these are hastily prepared by clinical or other professionals wishing to answer specific questions that they consider to be important. Unfortunately, the development and application of such questionnaires is often regarded as a matter of 'common sense' requiring little scientific consideration. However, in this area of research, common sense is commonly nonsense! In this article, I argue that many of the questionnaires patients are asked to complete in clinical practice and trials are of poor quality and collect information that is of scant relevance to the patient. In this respect, they are ultimately of limited value.

**Figure 1:**
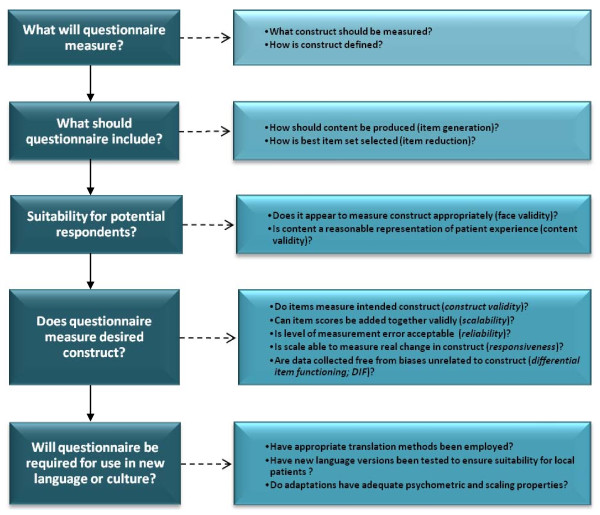
**Key considerations for patient-reported outcome questionnaire development**. The major factors that should be considered when selecting a patient-reported outcome measurement (PROM) for use in clinical studies are shown. These emphasise the importance of ensuring that the PROM addresses the required outcome, that it has been carefully developed and that all versions developed (including language adaptations) are of good quality.

Questionnaires used to elicit information from patients are now commonly referred to as patient-reported outcome measures (PROMs). A PROM is far more than a mechanism for gathering opinion. They are designed to measure a specific concept (that is, a construct) in a standardised way. Thus, they provide a means of quantifying qualitative information. In reality, there is a great deal of science involved in producing good-quality PROMs. Indeed, the PROM development process requires careful consideration of several key issues as set out in Figure 1.

When selecting a PROM, it is crucial that evidence is available to show that each of these key issues has been considered and addressed during instrument development and testing. Where measures are required for use in different languages or cultures, there are additional considerations: Have appropriate methods been employed to translate the questionnaire? Have new language versions been tested to ensure that they are both suitable for local patients and have adequate psychometric and scaling properties?

## What do PROMs measure?

Patient-reported outcome (PRO) is an umbrella term that covers a range of different types of outcome (see Table 1). Symptoms and functioning are clearly defined as impairments and disability in the International Classification of Impairment, Disability and Handicap [1]. Disability is now referred to as activity [2]. PROMs should not be confused with clinical rating scales, where a clinician completes a form to rate disease severity or treatment effects. The common link between PROMs is that they collect information directly from the patient without interpretation by clinicians or others [3-5]. However, this does not imply that all PROMs measure issues that are of concern or importance to the patient.

**Table 1:** Types of patient-reported outcome measures^a^

Type of PRO	Constructs assessed	Examples of coverage/domains
Symptoms	Impairment	· Pain · Fatigue · Anxiety · Depression · Incontinence
Functioning	Disability/activity	· Bathing · Dressing · Walking · Ability to work · Activities of daily living (such as personal care)
Health status (HRQL)	Combination of impairment, disability and, occasionally, some QoL	· Symptoms and functions as above
Quality of life	QoL	· Needs-based QoL
Utility^b^	Combination of impairment, disability or QoL	· Symptoms and functions as above · Activities of daily living (such as personal care) · Needs-based QoL

^a^PRO, patient-reported outcome; HRQL, health-related quality of life; QoL, quality of life; ^b^responses to the questionnaire are used to generate a perceived utility score.

Measures of symptoms, activity limitations, health status, health-related quality of life (HRQL) and quality of life (QoL) completed by patients are all examples of PROMs [3,6]. More recently, PROMs have also been used in clinical trials to address issues of patient satisfaction, compliance with treatment and treatment preferences. Each of these outcomes represents a distinct measurement construct and these should not be confused. Indeed, the term 'PRO' was coined in about 2000 specifically to avoid the misuse of, and the confusion surrounding, the term 'quality of life'. It had been (and occasionally still is) common practice for instrument developers to refer to any PROM as a measure of QoL, even where it was clearly designed to address a different outcome construct [7].

To summarise, PROMs that assess symptoms (that is, impairment) or functional limitations (such as disability or activity limitations) address issues that are of primary interest to the clinician, as these are most indicative of disease severity. HRQL measures are made up of scales that assess symptoms and activity limitations. In contrast, QoL scales determine outcomes that are of primary concern to the patient. Severe impairment or functional limitations may well also be of concern to the patient, but only where these affect QoL. QoL scales should provide a holistic assessment of the impact of disease and its treatment on the patient.

**Figure 2:**
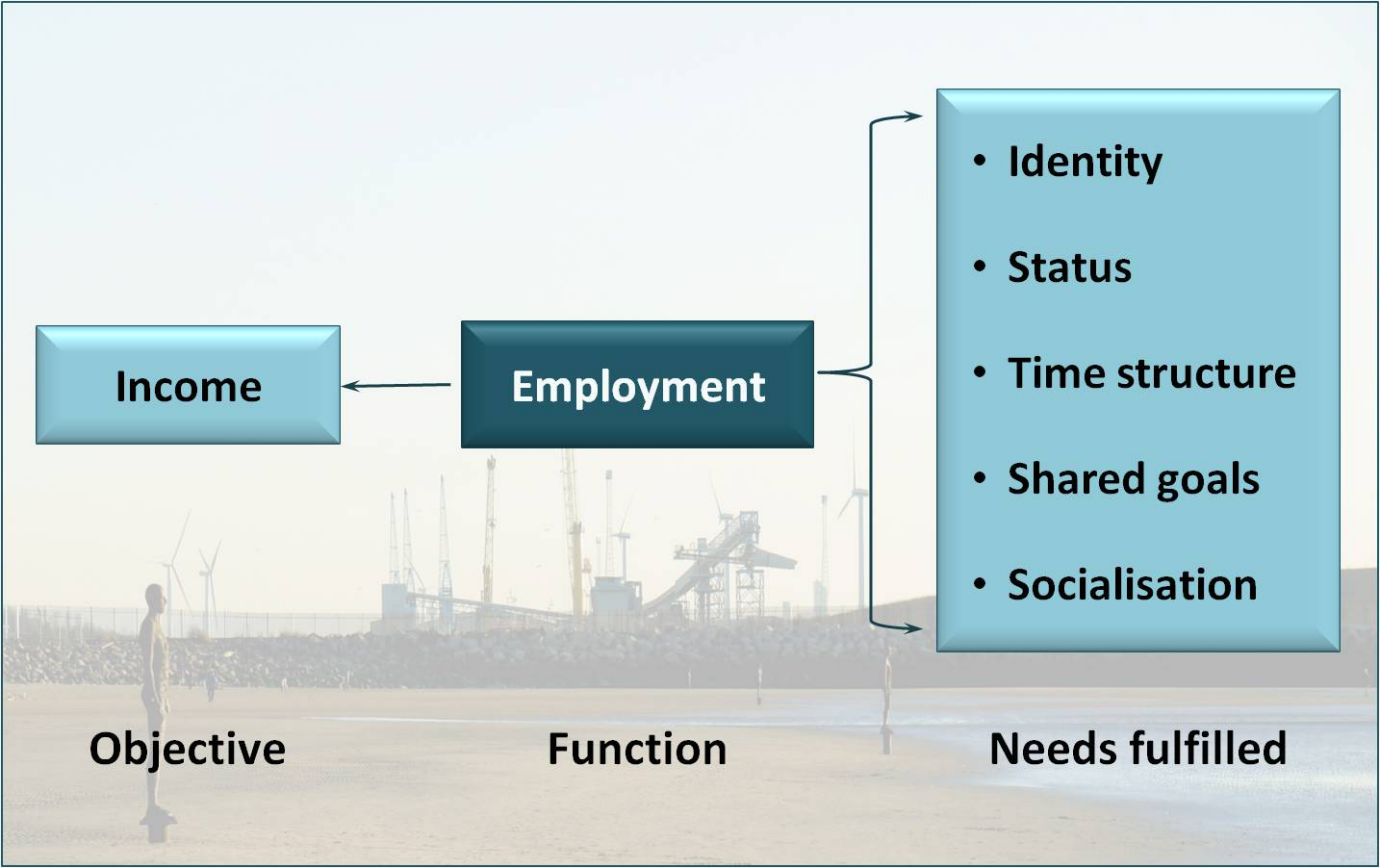
**Employment-related needs**. The relationships between function, objective and needs satisfaction are shown. Here employment is a function undertaken to obtain income. However, undertaking the function leads to the satisfaction of a range of needs (some of which are listed). Quality of life (QoL) is the result of satisfaction of the needs rather than earning an income *per se*.

Unfortunately, when describing PROMs, few authors state the model used to generate its content. Instead, it is common practice to describe a range of different constructs that should be measured. However, there is limited agreement about the specific constructs that should be assessed [8]. Of the measurement models described in the literature, the most widely applied QoL model is concerned with the extent to which disease and its treatment prevent an individual from meeting his or her needs [9-13]. This approach argues that individuals are driven or motivated by their needs and that the fulfilment of these provides satisfaction and a good QoL [9]. Consequently, QoL is good when most needs are fulfilled and poor when few needs are satisfied. Functioning is important only insofar as it permits need fulfilment. For example, employment has the objective of earning a salary, but it also leads to the fulfilment of a number of basic human needs (see Figure 2). Satisfaction of these needs leads to a good QoL.

Measures of satisfaction differ from HRQL and QoL, as they address the process of treatment rather than its outcome. These measures are concerned with factors such as acceptability of the drug and the quality of care.

Some PROMs, such as the EQ-5D [14] and the Health Utilities Index [15], can be used to generate preference or utility assessments. Patients' responses to these questionnaires can be converted to estimate the value of that person's life on a scale ranging from death (scored 0) through to perfect health (rated 1). As these PROMs consist of items enquiring about impairments and functional limitations, they are measures of HRQL. Such PROMs are referred to in this article as measures of utility, as they are widely used for this purpose in clinical trials. Recently, utility valuations have been derived from responses to disease-specific QoL instruments, providing more accurate measurement of this construct [16-20].

This article concentrates primarily on PROMs that assess more than a single symptom (such as pain or fatigue) or function (such as work or communication) that do not measure satisfaction or utility and that are used in clinical trials or for monitoring patients in clinical practise.

### Generic versus disease-specific PROs

Regardless of the construct assessed, a PROM may be generic or disease-specific. As its name implies, a generic instrument is intended to be used in any disease population. Some of the more widely known PROMs are generic. Examples include the Sickness Impact Profile (SIP) [21], the Nottingham Health Profile (NHP) [22], the Short Form 36 (SF-36) [23] and the EQ-5D [14]. Such instruments usually assess several domains and provide a profile of scores.

Traditionally, generic instruments were used to provide comparisons between diseases or to compare data with population normative values. However, the results of differential item functioning analyses show that such comparisons are scientifically flawed, as questionnaire items work in different ways with different patient groups [24-27]. This means that as generic measures cannot allow valid comparisons to be made between the impacts of different diseases or between healthy and diseased populations, they no longer have a clear role in measuring health outcomes.

A second major problem with generic instruments is that they are not designed to capture areas of concern to specific patient populations. This raises two issues. First, they are likely to include items that are irrelevant for certain patient groups. For example, questions that address physical functioning or bodily pain will only be relevant if they are a feature of the disease under study. Asking patients to answer questions that are irrelevant is likely to alienate respondents and increase the potential for missing or inaccurate responses. Second, they are likely to miss issues that are a specific feature of the disease under study. As a result, generic scales lack the responsiveness needed to measure change associated with effective treatment.

As a result of the acknowledgement of the problems with generic measures, they are no longer developed. They have partly been replaced by item-banking approaches whereby a subset of relevant items for a specific condition is selected to assess patients. The most widely used generic measures are relatively dated. The SIP and NHP were developed in the early 1970s. The five items included in the EQ-5D were taken from existing generic measures and so are of the same vintage. Most of the items in the SF-36 were adapted from instruments that had been used for 20 to 40 years previous to 1992 [23]. The way in which patients conceptualise their problems and the language with which they express themselves can change within a generation. Moreover, certain issues may become less important with time. For example, lack of mobility may be compensated for by advances in technology. Furthermore, the generic health status instruments have not benefited from improvements in test construction methodology and scaling techniques. Consequently, the reliability and responsiveness of the generic measures fall far short of what is required for instruments included in clinical trials.

Disease-specific questionnaires are developed to address those aspects of outcome that are important for a particular patient population. In the case of needs-based QoL measures, this is achieved by generating the items by means of qualitative interviews with relevant patients and by thoroughly testing the validity of the item set with new populations of patients. More complex analyses are also employed to ensure that all items actually assess the construct being measured [13,28]. Thus, for a well-developed measure, patients will only be asked questions that are relevant, meaningful and acceptable to them. Addressing the relevant areas of concern for the group under study maximizes respondent acceptability and minimises missing data. Consequently, disease-specific instruments possess greater potential for showing differences between competing therapies. A criticism that is often made of the use of disease-specific scales is the lack of comparability across diseases. This is a particular issue for reimbursement authorities, who are required to assess the comparative benefits of treatment reimbursement across disease areas. However, as noted above, the use of generic scales does not provide a valid basis for comparison across diseases. Recent advances in scaling theory are being applied to address this issue. It is now feasible to use disease-specific measures to make across-disease comparisons, providing the instruments are based on the same model of the construct measured.

### Use of PROMs in medicine

**Figure 3:**
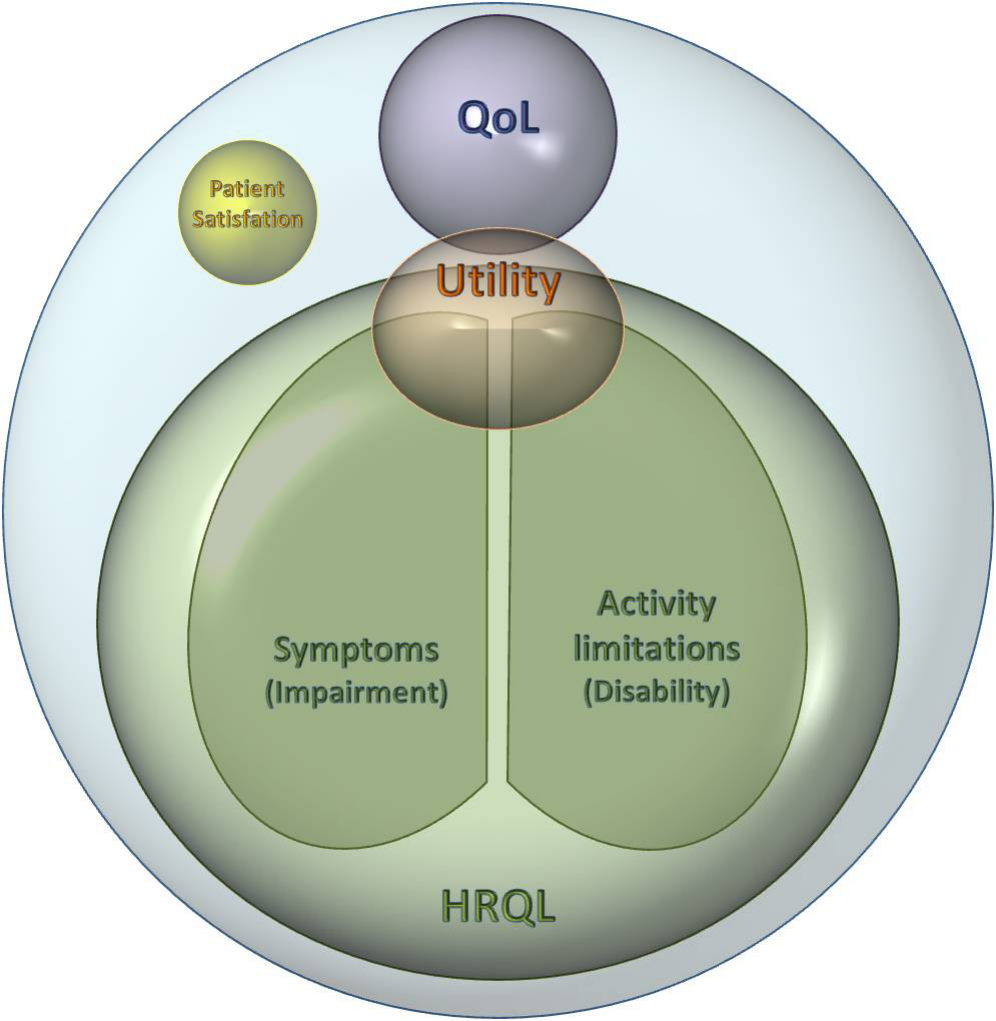
**Types of PROMs currently used in medical research**. The range of different types of patient-reported outcomes (PROs) is shown. The most commonly used PROMs assess symptoms and/or functional limitations. These are commonly referred to as health-related quality of life (HRQL) measures. The commonly used measures which generate utility values also ask about symptoms and/or functional limitations. Patient satisfaction is generally concerned with issues such as the process of treatment and relationships with clinical staff. QoL measures address need-fulfilment rather than symptoms and/or functional limitations.

PROMs have been used in a variety of ways in clinical practice and research. At the level of the individual patient, they can be used to assess disease severity and response to interventions. Here the measures can be used to help in decision making at the physician level. PROs are also widely used in clinical trials to determine whether an intervention is effective (for example, when evaluating treatments for pain) and also whether patients feel the benefit of treatments. Evidence provided by PROMs can thus aid decisions made by regulatory bodies regarding the utility of new products. Figure 3 shows schematically the different types of PROMs used in medicine. The diagram reflects the fact that most PROMs currently used assess HRQL rather than QoL or patient satisfaction. The assessment of QoL is relatively rare, despite the term being widely used in research reports and publications.

The widespread use of HRQL measures gives some cause for concern. First, the term itself is misleading and unhelpful insofar as it implies that QoL is being measured. Bradley [29] argued that 'clinicians may be misled into thinking that findings based on a [HRQL] instrument indicate that treatments do not damage QoL when all the data reveal is that treatments do not damage perceived health' (page 7). Indeed, the focus on HRQL provides a framework for assessing interventions predominantly from a clinical rather than a patient perspective. Second, HRQL scales do not necessarily address issues of primary concern to the patient. The focus of HRQL on the patient's ability to fulfil roles deemed 'normal' takes no account of the fact that patients with chronic disease adapt to their condition, often by replacing activities that they can no longer perform with others that are equally satisfying. Patients may give up functions that become problematic and take up other leisure activities to maintain their QoL. For example, while muscular degenerative disease patients may experience ambulatory problems, they can still remain independent and thus maintain a reasonable level of QoL through the use of a walking frame or wheelchair. HRQL measures are unable to cope with such adaptations, making it difficult for severely ill or disabled patients to show improvement even following effective interventions.

QoL is the primary outcome of relevance and importance to patients. When dealing with chronic diseases, the aim is frequently stated to be to improve the QoL of patients. This is particularly true where therapies cannot promise a cure or an extension to life. QoL is not intended to be an aid to diagnosis or a guide to the most appropriate intervention for a specific patient. However, its careful assessment should be able to determine which alternative interventions patients as a group would prefer within the context of a clinical trial. Despite this crucial role, there are several other requirements of clinical trials. For example, a product must be shown to improve objective health status and to be cost-effective. There are several chronic conditions for which steroid treatment improves QoL but does not necessarily improve the patient's health status in the long term.

The needs-based model of QoL resulted from analysing the transcripts from patient interviews conducted during the development of the Quality of Life in Depression Scale [9]. Figure 2 illustrates how the function of employment fits into the needs model. The objective purpose of employment is to earn money. However, being employed can lead to the satisfaction of a wide range of needs. Depressed patients who were unable to be employed reported problems with structuring their days, with identity and status and with reduced social interaction. Such needs can also be met in different ways, for example, by doing voluntary work or by joining sporting or interest clubs [30]. Research has shown that unemployed people who stay active in these ways are able to maintain their health [31].

The needs-based approach to QoL assessment has a number of advantages for measurement of the impact of disease and its treatment. Rather than asking directly about a function, it is possible to enquire about the needs that could be satisfied by that function. For example, questions about sexual performance are frequently left unanswered in questionnaires because of their irrelevance or unacceptability. The needs approach allows questions to be asked about needs related to sexual functioning that can also be satisfied in other ways, such as love, intimacy, touching and sharing with another person. The needs-based approach also copes well with patient adaptation. A chronically ill person can maintain a reasonable level of QoL by remaining independent through the use of aids and/or assistance. Patients who have activity limitations can still be shown to have a good QoL, as the concern here is the degree to which they can meet their needs, regardless of how this is achieved.

Measures developed using the needs-based approach are disease-specific (or could be more appropriately described as disease-relevant). This allows them to focus on the specific needs interfered with by the disease and hence makes them highly relevant and acceptable to the patient. As specific needs may be affected by different illnesses, it is possible to develop valid methods of making comparisons between the impacts of different diseases.

A further advantage of the needs-based measures is that they assess the single construct of need satisfaction, allowing the construction of unidimensional scales or indices of QoL. A major problem of HRQL measures is that they collect information on a range of different types of outcomes. Consequently, they provide a profile of scores (see, for example, the NHP [22], the SIP [21] and the SF-36 [23]). It is not possible to compare scores on the different sections of the profile, and it is certainly unacceptable simply to add together responses to the different sections to give a single score, although this is common practice in outcome measurement.

## Selecting and using PROMs for clinical trials and studies

The inclusion of poorly designed or inadequately targeted instruments in a clinical trial or study can have serious consequences. Furthermore, ethical questions are raised by asking patients to complete measures that are incapable of demonstrating treatment effects. It is strongly recommended that expert help is sought in selecting an appropriate PROM. Too often the choice is based on issues that are helpful rather than being of scientific importance. PROMs may be selected because they are commonly used, are used by a competitor or are available in a wide range of languages. While such factors can be helpful, they are minor compared with what the questionnaire actually measures and how well it does this.

When selecting a PROM, it is first necessary to determine the constructs that have to be assessed to meet the objectives of the study. Having done this, the next stage is to find PROs that measure these constructs well. It is not advisable to rely on, or to be limited to, the PROMs listed in databases such as OLGA [32] or the Patient-Reported Outcome and Quality of Life Instruments Database (PROQOLID) [33]. Such sources of information are often selective and/or omit important measures. Furthermore, they rely on test authors to provide information on the quality of the measures listed without providing any commentary on the acceptability of testing methods used or the appropriateness of the conclusions drawn. A thorough search of the medical literature should be made to find available measures and evaluate their suitability for use in the trial. This will often generate a host of potential PROMs that will vary considerably in terms of the care with which they were developed and their psychometric quality.

**Figure 4:**
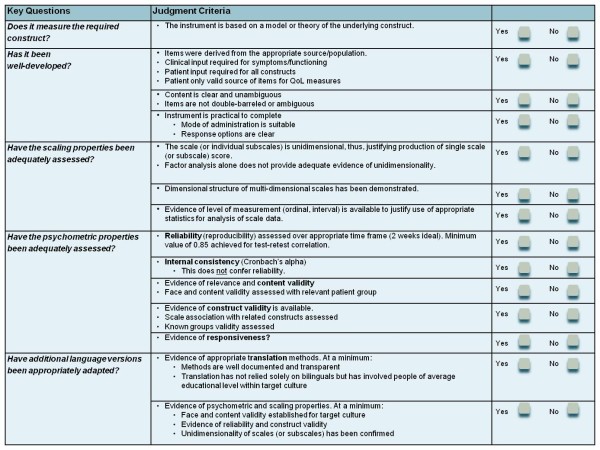
**Brief checklist for assessing the quality of PRO instruments**. The specific requirements of a good-quality PROM are shown. These qualities should be clearly reported in peer-reviewed publications. In many cases (including that of the most commonly employed PROMs), this information is not available. New instrument development methodologies, in particular the establishment of the scaling properties of a measure (item response theory), are essential to ensuring the quality of PROMs.

Selecting the most appropriate questionnaire requires consideration of several key quality standards. These cover the development processes, instrument scaling, psychometric properties and cultural translation and adaptation processes (Figure 4) [6]. These standards are described in detail in the Appendix.

It is increasingly common for trials to include PROMs. In some cases, they have been accepted as primary end points by the health authorities. However, these PROMs actually measure clinical end points (such as pain) that cannot be determined objectively. Indeed, the US Food and Drug Administration (FDA) prefers these types of PROs to be employed, as they appear to be uncomfortable with more subjective outcomes [34,35]. This contrasts with the European Medicines Agency (EMA), which welcomes QoL outcomes that describe the added benefits of new products [36]. Both the EMA and the FDA emphasise that outcome measures selected for a study should be well targeted to the specific patient population, which fundamentally rules out the use of generic PROMs. It is noticeable that both bodies now consider the most widely used PROM, the SF-36, to be unsuitable for making claims about the value of treatments. Indeed, this measure has such poor psychometric properties that it has never proved to be a valuable instrument for showing differences between active treatments (see, for example, [37] and [38]). Indeed, it has been shown that sample sizes of up to 20,000 per study arm would be required for SF-36 domains to be able to show such differences [39].

Where the instrument is used as a clinical end point in a trial and/or is intended to be used to support a product label claim or to provide information for inclusion in the Summary of Product Characteristics, it is necessary to agree in advance with the appropriate authorities that data collected with the measure will be acceptable to them. This generally involves providing a detailed briefing book. The briefing book must include information on how each item was generated and the reasons for rejecting items. Evidence is required for the whole testing procedure and the development and validation of all language versions of the measure to be used in the trial. Problems will occur with older measures, where such information is unlikely to be available and/or the development methodology was inadequate. Where a new measure is being developed for a specific trial, it is prudent to keep the authority informed at each stage of instrument development. The EMA now has a biomarker qualification system in operation that allows PRO instruments to be evaluated [40]. Once qualification has been achieved, the EMA will accept all data collected in a trial that uses the measure. The FDA has also issued a draft guidance document covering PRO instrument qualification [41].

Sufficient time should be allowed to ensure that the required language versions of a measure have been developed and validated (see below). Very often poor quality translations are produced, relying on simple forward-backward translation techniques rather than using an approach that involves relevant patients. Adapting measures appropriately is a time-consuming procedure that needs to be built into trial planning.

Once an instrument has been selected, it is crucial that its value and the reasons for its use are clear to everyone involved in the trial. If this is not done, data collection with the measure will be of debatable value. Staff involved in the trial at each centre will require training on the application of the measure and how to deal with problems that might arise.

## Development and validation of PRO measures

Where a search fails to identify a high-quality PROM for a trial, it will require that a new questionnaire be developed. However, planning for such an event is important, as the development process can be time-consuming, particularly if several language versions of the measure are required. I would argue strongly that the content for such a measure should be generated by means of one-to-one patient interviews, as the content should be relevant and acceptable to future patients.

There are four key stages in instrument development: (1) identification of the measurement model, (2) generation of questionnaire content, (3) content refinement and item reduction and (4) scaling and psychometric evaluation. These stages are summarised in Table 2[42-44].

**Table 2:** Development and validation of QoL measures

There are four key stages in instrument development:
▶ *Identification of measurement model*: QoL scales should be based on a stated model or theory of QoL.
▶ *Generation of questionnaire content*: Content of all QoL scales should be derived from interviews with relevant patients. Both the concerns and the wording used in the items should be generated during these interviews. Thirty to thirty-five interviews are usually sufficient to generate items. Qualitative analysis of the transcripts allows the construction of a QoL outcome model for the disease.
▶ *Content refinement and item reduction*: Content validity is assessed by comparing the issues covered by the items to the outcome model. Retained items should be clearly expressed, address only one issue, avoid duplication, be potentially capable of change and apply to all respondents. The draft measure should then be tested with a new set of patients to check comprehension, ability to answer the measure and ensure item relevance.
▶ *Scaling and psychometric evaluation*: Formal testing of dimensionality, reproducibility and construct validity should be achieved by means of a test-retest survey. In most European countries and North America, the survey can be conducted by post. A sample of 100 or more is preferable. It is strongly recommended that this stage should employ Item Response Theory techniques [42,43].

^a^QoL, quality of life.

### Adapting PRO measures

If the required language versions are known from the outset, instrument development should be conducted in parallel in these countries [45,46]. However, this information is rarely available, and it is more common for subsequent language adaptations to be required. Again, it is necessary to allow sufficient time for such adaptations to be produced, as the process can be time-consuming.

### Translation procedures

Translating PROMs is a complex task that cannot be undertaken lightly without the risk of producing poor-quality adaptations. It is commonly stated that forward-backward translation is the gold standard in translation methodology [47]. However, there is no evidence to support this view; it is merely a statement of belief. When such translation work was first handed to translators, test developers felt the need to assess the quality of the new version by some sort of 'scientific' method. This led to the introduction of forward-backward translation. However, such a methodology raises the hackles of translators, and not only because it casts inappropriate doubts on their abilities. If the translation is good, then the back-translation may well look nothing like the source questionnaire. Consequently, little information of value is obtained by conducting the backward translation, while misleading impressions can result. Instead, quality should be built into every stage of the translation procedure rather than checking it *a posteriori*.

Rather than relying on forward-backward translation, a dual-panel methodology has been developed and is now commonly employed (see Table 3). A recent study has shown that the 'dual panel' methodology produces translations that are more acceptable to patients in the new country than the use of forward-backward translation [48].

**Table 3:** Recommendations for the production of high-quality adaptations

The dual panel method is recommended for producing high-quality translations. The following recommendations are made:
Recruit 'translators' who currently live in the target country and whose command of English is good.
The meeting should be held in the country for which the measure is required.
Five to seven people enable fruitful discussion.
It is preferable to exclude professional translators.
An instrument developer should attend this meeting to explain the intent of the items and their specific meanings in the context of the questionnaire.
Inform the group of the model underlying the questionnaire, how it was developed, its design and its content and target audience.
Inform the group of the translation requirements (in particular accessibility and acceptability of wording).
The group should work as a team with a co-ordinator whose task is to check that none of the parameters are neglected (in particular, structural and metric aspects that could be overlooked).
Allow adequate time for the meeting to explore all issues fully.
Once the translated version of the instrument is agreed, have it assessed by a lay panel, again working as a group:
The coordinator involved in the first panel should work with this panel also to ensure that the original meaning of the items and the questionnaire structure are maintained.
The results of this meeting should be used to make final decisions about the wording of the questionnaire.
The whole procedure should be reported in detail, in particular explaining translation choices and changes made following lay panel testing. This not only provides information on the process undertaken but also constitutes a thorough final review.

It is important to remember that this is only the start of the adaptation process. The new translation should then be tested by means of face-to-face interviews with several relevant patients to ensure that the adapted version has face and content validity (known as 'cognitive debriefing'). Finally, the psychometric properties of the adapted questionnaire must be established with new patient samples. This requires a test-retest survey to be conducted for each new language version produced. Such retesting is rarely undertaken but is necessary to show that the new language version works in the same way as the original, evidence that is required by the FDA [35].

## Conclusions

The development, administration, analysis and adaptation of PROMs must be carried out by highly skilled specialists. Too often nonspecialists are given the tasks of determining which outcomes should be included in clinical studies and trials and how these should be measured. Unfortunately, this largely explains why few such studies provide useful data. Such a situation represents a waste of resources and the opportunity to show the benefits of expensive new products. A more professional approach to assessing PROs is needed. Of particular concern is the paucity of QoL studies undertaken, given that high-quality measures specific to several diseases are available [49].

Selecting the best PROMs for a trial should be given the same consideration as choosing clinical outcome measures. Too often PROMs are selected at too late a stage to allow required language adaptations to be produced. Consequently, less suitable measures are often selected. It is common for very expensive clinical trials to waste the opportunity to assess QoL or other PROs appropriately because of lack of planning or unwillingness to pay for the necessary development work. In reality, the cost of such work is minimal in comparison to the overall cost of the trial.

The development of PROMs is far from a commonsense procedure. Success is dependent on both expertise and experience. Table 4 lists some of the issues covered in this article. Many if not most of the points listed are counter to the commonsense view on outcome measurement and instrument development.

**Table 4:** A new common sense for patient-reported outcome assessment^a^

Do not rely on instrument databases for PRO identification and selection.
HRQL consists of symptoms, functions and limited aspects of the impact of these.
HRQL is very different from QoL.
The needs-based model of QoL is the most widely employed in medical research.
True QoL has rarely been measured in clinical studies and trials.
The content of QoL measures must be derived from relevant patients.
PROMs must be simple to administer, complete and score.
Simple two-point response formats are preferable to multiple response formats [43].
All PROMs used in clinical trials should be disease-specific.
Generic PROMs do not allow the impact of different diseases on patients to be compared.
Population norms for PROMs are invalid.
Think twice before selecting generic measures such as the EQ-5D to determine utility estimates, as they have limited psychometric quality.
QoL is a unidimensional construct.
Data collected using PROMs must be shown to be unidimensional.
Scores on subscales can rarely be added together to give a total score.
High reliability (reproducibility) is crucial to the accuracy of PROMs.
Forward-backward translation is a flawed methodology, creating unnecessary work.
Think carefully before using PROMs developed in the Western world in Asia and Africa.
Evidence is required of the scalability, reproducibility and construct validity of all language versions of PROMs used in a clinical trial.

^a^PRO, patient-reported outcome; PROM, patient-reported outcome measure; HRQL, health-related quality of life; QoL, quality of life.

Given the expressed desire of organizations such as the FDA and the EMA to be made aware of the benefits of treatment from the consumer's perspective and the need to convince payers of the added benefit of new treatments, it is to be hoped that more attention will be paid in future to the assessment of the effects of new interventions from the patient's perspective.

The science of PROMs is developing quickly. For too long, outdated generic HRQL measures such as the SF-36, NHP and EQ-5D have been relied on in clinical studies. It is now well understood that such measures are inadequate for showing change over time or the different impacts of alternative interventions. Greater emphasis is now placed on measurement models, disease-specific measurement and the application of Item Response Theory rather than Classical Test Theory. Well-developed measures are now generally of better quality and are more sensitive than many clinical outcome measures.

The development and use of PROMs have suffered from a lack of theory and poor basic development work for far too long. We have been willing to continue using the same poor generic PROMs because we are familiar with them, despite their age, lack of quality and inability to do the job for which they are intended. Given the cost of clinical trials and the importance of evaluating health services from the perspective of the patient, it is essential that the quality of PROMs improves. It is also time to reject the view that the only valid PROs are symptom scores and limited functional assessments mimicking clinical outcomes. It is important for PRO practitioners to argue strongly on behalf of the patient that we should also measure carefully those outcomes that really matter to them.

## Abbreviations

DIF: differential item functioning; EMA: European Medicines Agency; FDA: US Food and Drug Administration; HRQL: health-related quality of life; NHP: Nottingham Health Profile; PRO: patient-reported outcome; PROM: patient-reported outcome measure; QoL: quality of life; SIP: Sickness Impact Profile.

## Competing interests

The author is a co-developer of several disease-specific needs-based measures of quality of life. These are widely used commercially by pharmaceutical companies to evaluate patient-related outcome in clinical trials

## Authors' contributions

The article was prepared by SPM.

## Author's information

SPM is Director of Research at Galen Research. He has worked in the field of instrument development and adaptation since the 1970s. Since 1988, he and his team have created over 30 disease-specific, patient-reported outcome measures, many of which are considered to be the instrument of choice for clinical trials and for monitoring patients in clinical practise.

## Appendix

### Development and validation of patient-reported outcome measures

There are four key stages in instrument development: (1) identification of the measurement model, (2) generation of questionnaire content, (3) content refinement and item reduction and (4) scaling and psychometric evaluation.

### Identification of the measurement model

The requirement for a measurement model appears to be common sense: How else is it possible to decide which items to include in the measure? However, it is astounding how infrequently test developers report the measurement model that guided the development of their measurement instrument. Measures of symptoms or functioning may well be based on the World Health Organisation's International Classification of Functioning, Disability and Health classification of impairments and activity limitations, respectively [1,2]. A measure of quality of life (QoL) is likely to be based on the needs-based model of QoL [9]. The model employed should be reported in the instrument development publication to allow readers the opportunity to consider whether the measure employed is reasonable and practical.

### Generation of questionnaire content

The development of patient-reported outcome (PRO) instruments is a highly skilled activity best undertaken by specialists in measurement and psychometrics. It is particularly important that the content of these instruments is generated by researchers experienced in qualitative interviewing techniques. Content for all PRO measures (PROMs) should be derived from interviews with relevant patients (for QoL scales) or experts and/or patients (for measures of health-related quality of life). Thus, if a measure of QoL specific to endometriosis is required, the content will be derived from qualitative interviews conducted with women experiencing the problem. Such interviews are not intended to explore issues identified in the literature or by clinical experts. Both the relevant concerns and the wording used in the interview questions must be generated during these interviews. This is the most crucial stage of instrument development and must be carried out by skilled specialists. If good-quality questionnaire items are not identified, the resulting instrument will be poor.

The interviews, which may last several hours, should be audio-recorded and transcriptions produced. It is generally found that 30 to 35 interviews are sufficient to generate items for a scale. Additional interviews tend not to identify new issues of importance. Interviewees will generally raise specific functions that are problematic for them. The skill of the interviewer is to probe such responses carefully to understand how the patient's life is impaired by such restricted functioning. The needs-based model of QoL grew out of such probing in the development of the Quality of Life in Depression Scale [9]. Depressed patients who were unable to work reported problems with structuring their days, with identity and status and with reduced social interaction (see Figure 1).

Qualitative analysis of the transcripts allows the construction of a PRO model for the disease. This model will identify the issues and/or needs that are relevant for assessment of patients with the disease studied. The analysis will also identify potential items for inclusion in the measure. Where possible, it is preferable to keep the wording used by interviewees for the items, although minor changes may be necessary. Items are then best expressed as statements made by patients, such as 'I've lost interest in food' or 'I feel dependent on other people'. Stating the items in this form leads to a response format of 'yes' or 'no' or 'true' or 'not true'. This is a natural way of responding to items that should enquire into issues that are clear-cut. The application of modern psychometric models (such as Rasch analysis) indicates that increasing the number of possible responses for an item does not increase the sensitivity of the scale. Instead, the final set of items should each represent a different amount of the construct measured in the same way that the marks on a ruler denote different lengths.

### Content refinement and item reduction

Patient interviews will identify a large set of potential items. Content validity is assessed by comparing the issues covered by the items to the outcome model and other sources of information about the impact of the disease. The first stage of item reduction involves ensuring that items are clearly expressed. For example, they should address only one issue, avoid duplication, be potentially capable of change with effective treatment (for example, avoiding statements such as 'I worry that my illness will become worse') and apply to all respondents. Items that are not relevant are poor, as they lead to ambiguous responses.

### Scaling and psychometric evaluation

The next stage is to test the draft questionnaire with a new set of relevant patients by means of cognitive debriefing interviews. These items will explore interviewee's ability to understand and complete the measure and ensure that items are considered relevant. In this way, the face validity of the measure will be established. Changes in wording can still be made at this stage, and items can be removed or added as a result of the interviews.

Formal testing of the questionnaire for dimensionality, reproducibility and construct validity is then undertaken by means of a test-retest survey. In most European and North American countries, the survey can be conducted by post. While test-retest reliability (reproducibility) can be assessed with a sample of around 50, the need to determine the dimensionality of the scale means that a sample of 100 or more is preferable.

## Pre-publication history

The pre-publication history for this paper can be accessed here:

http://www.biomedcentral.com/1741-7015/9/86/prepub

## References

[B1] World Health Organization International Classification of Impairments, Disabilities and Handicaps: A Manual of Classification Relating to Consequences of Disease 1980 Geneva: World Health Organization

[B2] World Health Organization International Classification of Functioning, Disability and Health 2001 Geneva: World Health Organization

[B3] Acquadro C Berzon R Dubois D Leidy NK Marquis P Revicki D Rothman M PRO Harmonization Group Incorporating the patient's perspective into drug development and communication: an ad hoc task force report on the patient-reported outcomes (PRO) harmonization group meeting at the Food and Drug Administration, February 16, 2001 Value Health 2003 5 522 531 10.1046/j.1524-4733.2003.65309.x 14627058

[B4] Burke LB Kennedy DL Miskala PH Papadopoulos EJ Trentacosti AM The use of patient-reported outcome measures in the evaluation of medical products for regulatory approval Clin Pharmacol Ther 2008 84 281 283 10.1038/clpt.2008.128 18580868

[B5] EMEA Committee for Medicinal Products for Human Use (CMHP) Reflection paper on the regulatory guidance for the use of health-related quality of life (HRQL) measures in the evaluation of medicinal products Doc Ref EMEA/CHMP/EWP/139391/2004 2005 London: European Medicines Agency http://www.ema.europa.eu/docs/en_GB/document_library/Scientific_guideline/2009/09/WC500003637.pdf

[B6] Doward LC McKenna SP Defining patient-reported outcomes Value Health 2004 7 Suppl 1 S4 S8 15367236 10.1111/j.1524-4733.2004.7s102.x

[B7] Burke L Acceptable evidence for pharmaceutical advertising and labeling DIA Workshop on Pharmacoeconomics and Quality of Life Labeling and Marketing Claims, October 3, 2000, New Orleans, LA 2000 Horsham, PA: Drug Information Association

[B8] Doward LC McKenna SP Rajagopalan R, Sheretz EF, Anderson RT Evolution of quality of life assessment Care Management of Skin Diseases: Life Quality and Economic Impact 1997 New York: Marcel Dekker 9 33

[B9] Hunt SM McKenna SP The QLDS: A scale for the measurement of quality of life in depression Health Policy 1992 22 307 319 10.1016/0168-8510(92)90004-U 10122730

[B10] Doyal L Gough I A Theory of Human Need 1991 Basingstoke, UK: Macmillan

[B11] Hyde M Wiggins RD Higgs P Blane DB A measure of quality of life in early old age: the theory, development and properties of a needs satisfaction model (CASP-19) Aging Ment Health 2003 7 186 194 10.1080/1360786031000101157 12775399

[B12] Hornquist JO The concept of quality of life Scand J Soc Med 1982 10 57 61 7178870 10.1177/140349488201000204

[B13] Gilworth G Chamberlain MA Bhakta B Haskard D Silman A Tennant A Development of the BD-QoL: a quality of life measure specific to Behçet's disease J Rheumatol 2004 31 931 937 15124253

[B14] Rabin R de Charro F EQ-5D: A measure of health status from the EuroQol Group Ann Med 2001 33 337 343 10.3109/07853890109002087 11491192

[B15] Macran S Weatherly H Kind P Measuring population health: a comparison of three generic health status measures Med Care 2003 41 218 231 12555050 10.1097/01.MLR.0000044901.57067.19

[B16] Doward LC McKenna SP Meads DM Ratcliffe J Whalley D Langley PC Investigation into the feasibility of deriving relative and absolute utility from the Recurrent Genital Herpes Quality of Life Questionnaire (RGHQoL) Value Health 2002 5 573 574

[B17] McKenna SP Ratcliffe J Meads DM Brazier JE Development and validation of a preference based measure derived from the Cambridge Pulmonary Hypertension Outcome Review (CAMPHOR) for use in cost utility analyses Health Qual Life Outcomes 2008 6 65 10.1186/1477-7525-6-65 18718016 PMC2546377

[B18] Meads DM McKenna SP Doughty N Das C Gin-Sing W Langley J Pepke-Zaba J The responsiveness and validity of the CAMPHOR Utility Index Eur Resp J 2008 32 1513 1519 10.1183/09031936.00069708 18768576

[B19] Mavranezouli I Brazier JE Young TA Barkham M Using Rasch analysis to form plausible health states amenable to valuation: the development of CORE-6D from a measure of common mental health problems (CORE-OM) Qual Life Res 2011 20 321 333 10.1007/s11136-010-9768-4 20972629

[B20] McTaggart-Cowan HM Marra CA Yang Y Brazier JE Kopec JA FitzGerald JM Anis AH Lynd LD The validity of generic and condition-specific preference-based instruments: the ability to discriminate asthma control status Qual Life Res 2008 17 453 462 10.1007/s11136-008-9309-6 18274882

[B21] Bergner M Bobbit RA Kressel S Pollard WE Gilson BS Morris JR The Sickness Impact Profile: conceptual formulation and methodology for the development of a health status measure Int J Health Serv 1976 6 393 415 10.2190/RHE0-GGH4-410W-LA17 955750

[B22] Hunt SM McEwen J McKenna SP Measuring Health Status 1986 London: Croom Helm

[B23] Ware JE Sherbourne CD The MOS 36-Item Short-Form Health Survey (SF-36) Med Care 1992 30 474 483 1593914

[B24] Nijsten T Meads DM McKenna SP Dimensionality of the Dermatology Life Quality Index (DLQI): a commentary Acta Dermato-Venereologica 2006 86 284 285 10.2340/00015555-0075 16710607

[B25] Tennant A Penta M Tesio L Grimby G Thonnard JL Slade A Lawton G Simone A Carter J Lundgren-Nilsson A Tripolski M Ring H Biering-Sørensen F Marincek C Burger H Phillips S Assessing and adjusting for cross-cultural validity of impairment and activity limitation scales through differential item functioning within the framework of the Rasch model: the PRO-ESOR project Med Care 2004 42 1 Suppl I37 I48 14707754 10.1097/01.mlr.0000103529.63132.77

[B26] Bjorner JB Pejtersen JH Evaluating construct validity of the second version of the Copenhagen Psychosocial Questionnaire through analysis of differential item functioning and differential item effect Scand J Public Health 2010 38 3 Suppl 90 105 10.1177/1403494809352533 21172775

[B27] Chien CW Brown T McDonald R Rasch analysis of the assessment of children's hand skills in children with and without disabilities Res Dev Disabil 2011 32 253 261 10.1016/j.ridd.2010.09.022 21041063

[B28] Keenan AM McKenna SP Doward LC Conaghan PG Emery P Tennant A OAQoL: the development and validation of a quality of life instrument for osteoarthritis Arthritis Rheum 2008 15 841 848 10.1002/art.23714 18512719

[B29] Bradley C Importance of differentiating health status from quality of life Lancet 2001 357 7 8 10.1016/S0140-6736(00)03562-5 11197385

[B30] Jahoda M Employment and Unemployment: A Social Psychological Analysis 1982 Cambridge, UK: Cambridge University Press

[B31] Fryer DM McKenna SP Fineman S The laying off of hands Unemployment: Personal and Social Consequences 1987 London: Tavistock 47 73

[B32] Erickson P Taeuber RC Scott J Operational aspects of quality-of-life assessment: choosing the right instrument Pharmacoeconomics 1995 7 39 48 10.2165/00019053-199507010-00005 10155292

[B33] Emery MP Perrier LL Acquadro C Patient-Reported Outcome and Quality of Life Instruments Database (PROQOLID): frequently asked questions Health Qual Life Outcomes 2005 3 12 10.1186/1477-7525-3-12 15755325 PMC555954

[B34] Patrick DL Burke LB Powers JH Scott JA Rock EP Dawisha S O'Neill R Kennedy DL Patient-reported outcomes to support medical product labeling claims: FDA perspective Value Health 2007 10 Suppl 2 S125 S137 17995471 10.1111/j.1524-4733.2007.00275.x

[B35] US Department of Health and Human Services US Food and Drug Administration Guidance for Industry: Patient-Reported Outcome Measures. Use in Medical Product Development to Support Labeling Claims 2009 Washington, DC: US Food and Drug Administration

[B36] European Medicines Agency Committee for Medicinal Products for Human Use (CMHP) Reflection Paper on the Regulatory Guidance for the Use of Health-Related Quality of Life (HRQL) Measures in the Evaluation of Medicinal Products 2005 London: European Medicines Agency

[B37] Güthlin C Walach H Structural equation modeling to test the construct validity of the second-order factor structure Eur J Psychol Assess 2007 23 15 23 10.1027/1015-5759.23.1.15

[B38] Horner-Johnson W Krahn GL Suzuki R Peterson JJ Roid G Hall T RRTC Expert Panel on Health Measurement Differential performance of SF-36 items in healthy adults with and without functional limitations Arch Phys Med Rehabil 2010 91 570 575 10.1016/j.apmr.2009.12.015 20382289

[B39] Dixon P Heaton J Long A Warburton A Reviewing and applying the SF-36 Outcomes Briefing 1994 4 3 25

[B40] European Medicines Agency Committee for Medicinal Products for Human Use (CMHP) Draft Biomarkers Qualification: Guidance to Applicants 2008 London: European Medicines Agency

[B41] US Food and Drug Administration Qualification Process for Drug Development Tools http://www.fda.gov/downloads/Drugs/GuidanceComplianceRegulatoryInformation/Guidances/UCM230597.pdf

[B42] Tennant A Gonaghan PG The Rasch measurement model in rheumatology: What is it and why use it? When should it be applied, and what should one look for in a Rasch paper? Arthritis Rheum 2007 57 1358 1362 10.1002/art.23108 18050173

[B43] Pallant JF Tennant A An introduction to the Rasch measurement model: An example using the Hospital Anxiety and Depression Scale (HADS) Br J Clin Psychol 2007 46 1 18 10.1348/014466506X96931 17472198

[B44] De Jong Z van der Heijde D McKenna SP Whalley D The reliability and construct validity of the RAQoL: A rheumatoid arthritis-specific quality of life instrument Br J Rheumatol 1997 36 878 883 10.1093/rheumatology/36.8.878 9291857

[B45] Doward LC McKenna SP Kohlmann T Niero M Patrick D Spencer B Thorsen H The international development of the RGHQoL: a quality of life measure for recurrent genital herpes Qual Life Res 1998 7 143 153 10.1023/A:1008857426633 9523496

[B46] McKenna SP Doward LC Alonso J Kohlmann T Niero M Prieto L Wíren L The QoL-AGHDA: an instrument for the assessment of quality of life in adults with growth hormone deficiency Qual Life Res 1999 8 373 383 10.1023/A:1008987922774 10472170

[B47] Wild D Grove A Martin M Eremenco S McElroy S Verjee-Lorenz A Erikson P Principles of good practice for the translation and cultural adaptation process for patient reported outcomes (PRO) measures: report of the ISPOR task force for translation and cultural adaptation Value Health 2005 8 94 104 10.1111/j.1524-4733.2005.04054.x 15804318

[B48] Hagell P Hedin PJ Meads DM Nyberg L McKenna SP Effects of method of translation of patient-reported health outcome questionnaires: a randomized study of the translation of the Rheumatoid Arthritis Quality of Life (RAQoL) instrument for Sweden Value Health 2010 13 424 430 10.1111/j.1524-4733.2009.00677.x 20070642

[B49] McKenna SP Doward LC Meads DM Patrick D Tennant A Summary of needs-based quality of life instruments Value Health 2004 7 Suppl 1 S39 S40 10.1111/j.1524-4733.2004.7s109.x 15367243

